# Synthesis and Antitumor Activity of New Group 3 Metallocene Complexes

**DOI:** 10.3390/molecules22040526

**Published:** 2017-03-28

**Authors:** Angelamaria Caporale, Giuseppe Palma, Annaluisa Mariconda, Vitale Del Vecchio, Domenico Iacopetta, Ortensia Ilaria Parisi, Maria Stefania Sinicropi, Francesco Puoci, Claudio Arra, Pasquale Longo, Carmela Saturnino

**Affiliations:** 1 Department of Science, University of Basilicata, Viale dell'Ateneo Lucano 10, Potenza 85100, Italy; acaporale@unisa.it; 2 SSD Sperimentazione Animale, Istituto Nazionale Tumori, IRCCS, “Fondazione G. Pascale”, Via Mariano Semmola, Napoli 80131, Italy; palma.giuseppe@icloud.com (G.P.); vitale_84@hotmail.it (V.D.V.); 3 Department of Biology and Chemistry, University of Salerno, Via Giovanni Paolo II, 132, Fisciano 84084, Italy; amariconda@unisa.it (A.M.); plongo@unisa.it (P.L.); 4 Department of Pharmacy, Health and Nutritional Sciences, University Calabria, Via Pietro Bucci, Arcavacata di Rende 87036, Italy; domenico.iacopetta@unical.it (D.I.); ortensiailaria.parisi@unical.it (O.I.P.); s.sinicropi@unical.it (M.S.S.); francesco.puoc i@unical.it (F.P.)

**Keywords:** breast cancer, prostate cancer, metallocene group 3, metal complexes, cytotoxic activity, cell membrane permeability

## Abstract

The quest for alternative drugs with respect to the well-known *cis*-platin and its derivatives, which are still used in more than 50% of the treatment regimens for patients suffering from cancer, is highly needed. In this context, organometallic compounds, which are defined as metal complexes containing at least one direct covalent metal-carbon bond, have recently been found to be promising anticancer drug candidates. A series of new metallocene complexes with scandium, yttrium, and neodymium have been prepared and characterized. Some of these compounds show a very interesting anti-proliferative activity in triple negative breast cancer cell line (MDA.MB231) and the non-hormone sensitive prostate cancer cell line (DU145). Moreover, the interaction of some of them with biological membranes, evaluated using liposomes as bio-membrane mimetic model systems, seems to be relevant. The biological activity of these compounds, particularly those based on yttrium, already effective at low concentrations on both cancer cell lines, should be taken into account with regard to new therapeutic approaches in anticancer therapy.

## 1. Introduction

After the remarkable effectiveness demonstrated by *cis*-platinum [*cis*(NH_3_)_2_PtCl_2_] as an anticancer drug, mainly in the clinical treatment of ovarian, testicular, and head and neck cancers [1,2,3], scientists have developed considerable studies to obtain new organometallic compounds with less toxic effects [4]. 

The *cis*-platinum and its derived molecules, as oxaliplatin and carboplatin, are still in use in more than 50% of cancer treatment; on the other hand, these platinum compounds suffer from two major drawbacks: they are ineffective against platinum-resistant cancer, and have serious side effects, such as nephrotoxicity and neurotoxicity [5,6]. 

Platinum complexes have labile *cis*-chloride groups or similar ligands that enter into the cells by diffusion, here they are converted to the active form; this species is not determined with great sureness. It is believed that the most active species is the monohydrate platinum complex, even if the most common one is the di-hydrate form. The active *cis*-platin compound interacts with many types of proteins involved in DNA replication and cell division, but the main target is the DNA double strand [7,8,9,10,11].

An important aspect in cancer research is represented by achieving new molecules with lower toxicity and higher selectivity towards cancer cells with respect to the drugs already used in clinical treatment [4,12]. 

In this context, organometallic compounds, defined as metal complexes containing at least one direct covalent metal-carbon bond including those of group 3, have recently been highlighted as good anticancer candidates for their significant biological activity [13,14,15,16,17,18,19,20,21,22,23,24,25,26,27,28].

Many of the pharmacological properties of the group 3 metals are due to the ability to replace calcium ions (Ca^2+^) in biological molecules and strongly bond water molecules; furthermore, they can also substitute metal ions Mg^2+^, Mn^2+^, and Fe^3+^ in biological effectors involved in cell mechanisms [13,14]. These features lead to modifying many cellular processes, for example, inhibiting or activating calcium-dependent enzymes. This different regulation depends on the role of calcium in the native enzyme: if calcium plays a catalytic role, its substitution by an ion of group 3 leads to the enzyme deactivation, and the degree of inhibition depends on the ionic radius of the metal. Instead, if Ca^2+^ plays a structural role, the replacing with a M^3+^ ion preserves the function of the effector [13,14]. In this way the antitumor action of group 3 metal complexes is carried out through the positive or negative regulation of cell cycle effectors.

The group 3 metal and lanthanide complexes bound with coumarin, show higher activity in various tumor models than the corresponding inorganic salts [15,16,17,18]. Moreover texaphyrins, lanthanide complexes with monoanionic macrocyclic ligands containing five nitrogen atoms coordinated to the central core, are approved for clinical trials [19,20], as well as the series of cerium (III) with bipyridyl or phenanthroline ligands that show anti-proliferative activity against tumor cell lines [21,22]. 

Recently, the cytotoxic and anti-proliferative activity of several new group 3 compounds has been evaluated: lanthanides with heterocyclic ligands (tridentate monoanionic quinoline-phenoxy-amine, quinoline-phenoxyimine, and ansa-monocyclopentadienyl-immino-pyridine) [23,24,25,26,27], and also complexes with heteroscorpionate group 3 (i.e., *N*,*N*′-dicyclohexyl-2,2 *bis*-(3,5-dimethyl-pyrazol-1-yl)- acetamidinate). All of these molecules have proven to be good candidates for the synthesis of a wide range of stable compounds of group 3 and lanthanide derivatives with anti-proliferative activity [28]. 

The metals with cyclopentadienyl or cyclopentadienyl-derivative ligands instead need other detailed studies because they showed a strong anti-proliferative effect on cancer with a high grade of cytotoxicity [29,30,31,32,33,34].

The complexes of titanium (Group 4) with those of the third group metals may have different mechanisms of action, leading to a completely different specificity and selectivity.

The selected ligands have the ability to coordinate the metal center in a bi-dentate or mono-dentate manner. The latter type of coordination allows the ligand to give additional interactions with target molecules. Moreover, we synthesized both mono-cyclopentadienyl and bis-cyclopentadienyl group 3 metal complexes because, as observed with titanium derivatives, half-metallocenes and metallocenes may exert different activities [28,29,30,31]. 

In this paper, we report the synthesis and characterization of several group 3 metal complexes, selected among those who have already proved ability to stabilize the cation titanium by intramolecular or intermolecular coordination, and the data about their potential anti-proliferative activity in triple negative breast cancer and prostate cancer cell lines. Moreover we also show the interaction between some of the synthesized metal complexes and biological membranes, which often act as barriers in the physiological processes of absorption and distribution of therapeutic agents into the body, with a remarkable effect on the pharmacological effectiveness [35,36]. 

## 2. Results and Discussion

### 2.1. Chemistry

The group 3 metals have significant differences in atomic radius and electronegativity and this parameter could influence their antitumor activity; therefore, we synthesized scandium, yttrium, and neodymium complexes. 

**Figure 1:**
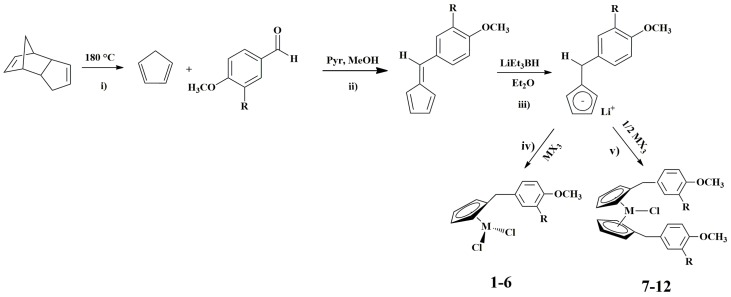
Synthetic route for the preparation of compounds **1**–**12**.

Synthesis of group 3 and lanthanide metal complexes was carried out in four steps in an inert nitrogen atmosphere, as reported in the Figure 1.

(i) Cyclopentadiene was obtained by a retro Diels–Alder of dicyclopentadiene at high temperature (180 °C).
(ii) The synthesis of fulvene pro-ligands by reaction of cyclopentadiene with suitable aromatic aldehyde.
(iii) Lithium salts of ligands were synthesized by reacting the appropriate fulvene with LiEt_3_BH (super-hydride), in dry diethyl ether. The reaction is a nucleophilic addition of a hydride to the double bond 5–6 of fulvene. We used a slight excess with respect to the stoichiometric amount of super-hydride. The choice of the Li(Et)_3_BH as reducing agent derived from its high selectivity. In fact, the double bond 5–6 of fulvene had a high polarity due to the inductive effect of the methoxy group bonded to benzene. By this increase of the polarity, the reducing agent attacked the double bond 5–6 and not the dienic component of fulvene [28,29,30].
(iv) Synthesis of group 3 and lanthanide complexes were carried out in THF at −78 °C, by the reaction of a chosen lithium salt of ligands with the suitable stoichiometric amount of metal(III) halide, in order to obtain half- and metallocene derivatives and, consequently, to evaluate the influence of these lipophilic groups on the biological activity of the compounds.

All of the synthesized complexes were purified following common procedures and isolated in good yields. Elemental analysis (C, H) were in agreement with the proposed formulae. ^1^H COSY experiments allowed the assignment of all the proton resonances of the ^1^H-NMR spectra, whereas DEPT experiments were useful for the attribution of ^13^C-NMR signals. The synthesized compounds were also characterized by mass spectrometry and elemental analysis. The set of these data (see Result and Discussion) allowed us to have an unambiguous structural determination, as reported in Figure 1 and Table 1.

#### Hydrolysis Stability

The hydrolysis stability of the six half-metallocene (**1**–**6**) and of six metallocene complexes (**7**–**12**) has been determined in aqueous solution, 90% *d*_6_-DMSO by ^1^H-NMR spectroscopy, in order to correlate the chemical stability and coordination chemistry of these complexes with their observed cytotoxic activity. Since we can expect that rapid hydrolysis of the leaving group (-Cl) and cyclopentadienyl ligands could give biologically-inactive species, an active species could be generated if the Cp rings remain metal-bound. Hydrolysis of aromatic rings of **1**–**6** and **7**–**12** complexes was evaluated by integration of two signals of protons of cyclopentadienyl bonded to metal, as to newly-formed multiples of substituted cyclopentadiene. All of the complexes show high hydrolytic stability; in fact, the cyclopentadienyl rings of complexes are hydrolyzed for less than 5% after 24 h. These data provide sufficient evidence that the presence of coordinating groups on the aryl substituent of the cyclopentadienyl are effective for the stabilization of the complexes. Therefore, these coordinating groups might be fundamental to increase their biological effectiveness.

### 2.2. Biological Activity

**Figure 2:**
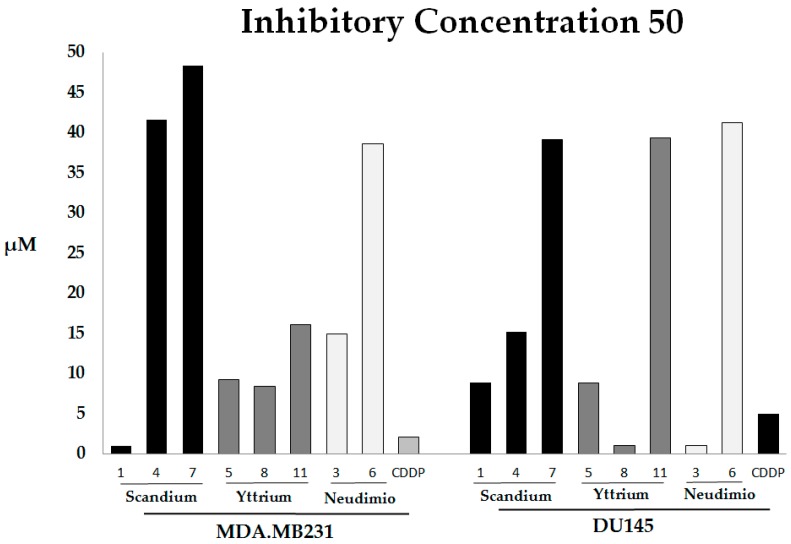
The cytotoxic activity of compounds was evaluated as IC_50_ (μM) responses from 5 μM to 100 μM of compounds **1**–**2** in MDA.MB231, human triple negative breast cancer and DU145, human non-hormone sensitive prostate cancer, defined by the MTT assay. Cell growth was measured as the percentage of cells treated with different concentrations, distinct compounds respect to cells treated for 48 h. The IC_50_ of **1**, **3**, and **8** are under the first value of tested concentration and for this reason it is not determined numerically; we show an approximated value.

In order to assess the cytotoxic effects on cancer cell proliferation of novel synthesized complexes, we treated MDA.MB231 and DU145 with each compound, testing concentrations in a range from 5 μM to 100 μM. The obtained data have been statistically analyzed in order to assess the concentration able to inhibit the 50% of cell growth respect to the untreated control (IC_50_ value). Several nonlinear functions have been commonly used to estimate the IC_50_ value, finding a relation between the drug concentration, on the *X* axis, and the percentage of survival at this concentration, on the *Y* axis. We propose a statistical nonlinear model, for a better evaluation of the dose response relationship between our variables. In our experiments, we apply a polynomial fitting curve using local regression; this is a least squares method which minimizes the error between the original data and the values predicted by the equation. The goodness of fit has been evaluated with the determination of an R^2^ value which is higher than 0.789 in all models of IC_50_ assessment of the described compounds, on both adopted cell lines, (0.789 < R^2^ < 0.987). In Figure 2 we report the cytotoxic activity and in Table 2 the IC_50_ values of active compounds on both cell lines from the MTT assay data processing.

All synthesized compounds show an inhibition growth activity (see Supplementary Materials) but compounds **10**, **2**, **9**, and **12** show a low efficiency yet in the highest concentrations. The yttrium complexes seem more efficient already at very low concentrations; in fact, the IC_50_ value for **5** and **8** is less than 5 μM. 

Our results show an impaired rate of growth inhibition by different compounds on the same cell line, but also by the same compound on different cell lines. The highest inhibition effect is made by compound **1**, on MDA.MB231, and by **3** and **8** on DU145 (the IC_50_ of these molecules is under the first value of the tested concentration and, for this reason, it is not determined numerically). Each of these molecules is comprised of a different metal core: **1** has the best activity amongst scandium compounds, **3** amongst neodymium compounds, and **8** amongst yttrium compounds. We can assess that, on the MDA.MB231 cell line, all of the compounds with yttrium show a good inhibition effect, metallocene as well as half-metallocene. Furthermore, the scandium-based compounds do not show a significant effect, except for **1**. Finally, the neodymium based compounds have a mild effect. On the DU145 cell line we observe a wide spectrum of IC_50_ referring to the same class of compounds.

The differences between the three series of synthesized complexes are certainly indicative of the very complex mechanisms of cell growth inhibition, closely related to the molecule structures and not only to the ligand or to metal ion separately. From a structural point of view the data analysis shows that, among the scandium compounds, the most active are the two half-metallocenes with the **1** which shows the best IC_50_ value on the MDA.MB231, as well as on DU145. The significant activities of the two half-metallocenes of scandium, compared with two scandiocenes, might be due to the small atomic radius of metal ion that could allow intramolecular coordination of the methoxy groups of the ligands in the case of half-scandiocenes, with a gain of stability of the complexes. This coordination may not be possible in the case of scandiocenes because of steric hindrance of ligands. 

The yttrium complexes with better activity, on both tested cell lines, were the half-metallocene **5** and the metallocene **8**. In this case, it seems that the inhibitor activity may be related to the presence of two methoxy groups: both on one aryl group in **5** or one for each aryl group in **8**. Clearly, for the stabilization of yttrium complexes, the coordination of two methoxyl groups to the metal ion core is required. The complexes of neodymium are not particularly active, except for **3**, which shows a strong activity, probably due to its lower electronegativity, than scandium and yttrium, which do not allow the intramolecular coordination of the methoxy groups, for which the complexes turn out to be less stable [37].

### 2.3. Evaluation of the Drug-Membrane Interaction

In our experiments, we used liposomes as mimetic models able to reproduce the anisotropic properties of cell membranes. In this way, we investigated the interaction between synthesized metal complexes and biological membranes, evaluating the cell permeability, also considering the electrostatic interaction. Based on these considerations, the use of this kind of membrane model is more reliable than the octanol/water partitioning to evaluate the lipophilicity of a drug.

The ability of a drug to interact with bio-membranes affects its biological properties in terms of both therapeutic and toxic effects. Therefore, the evaluation of the partitioning behavior between lipid and water phases, throughout the quantification of the partition coefficient (kp), allows to predict the ability of the therapeutic agent to pass across the membrane to estimate its in vivo activity. The partition coefficient (kp), which is considered a useful parameter for the design and development of new effective antitumor agents, of compounds **8** and **4** was evaluated by the ultrafiltration method. After the ultrafiltration process, drug concentrations in the aqueous phase were quantified by UV-VIS spectrophotometry and the obtained kp values were 1700 for **8** and 1200 for **4**. The experimental results indicate that both anticancer agents tested have shown a relevant membrane permeability.

## 3. Materials and Methods 

### 3.1. General Procedure

All manipulations were carried out under oxygen- and moisture-free atmosphere, using Schleck techniques or working in an MBraun MB 200 glove-box (Daimlerstraße 29-31 D-76316 Malsch, Germany).

### 3.2. Solvents

All the solvents were thoroughly deoxygenated and dehydrated under argon by refluxing over suitable drying agents; while NMR deuterated solvents (Euriso-Top products, Parc des Algorithmes, Bâtiment Homère Route de l'Orme F-91194 Saint-Aubin Cedex, France) were kept in the dark over molecular sieves. 

### 3.3. Reagents

All chemicals were obtained from Sigma-Aldrich (Sigma Aldrich SRL Via Gallarate. 154 I-20151 Milano, Italy) and used without further purification. Cyclopentadiene was obtained by freshly cracked dicyclopentadiene. 

### 3.4. Characterization Techniques

**^1^**H-NMR, homodecoupled ^1^H-NMR, ^1^H COSY, and ^13^C-NMR spectra were recorded on a Bruker Avance 300 Spectrometer (Bruker Co., Billerica, MA, USA) operating at 300 MHz (^1^H) and 75 MHz (^13^C). Chemical shifts referring to tetramethylsilane were used as the internal standard.

### 3.5. Cell Lines

The MDA-MB-231 tumor cell line of triple negative breast human cancer was purchased from American Type Culture Collection (ATCC® HTB-26™, Manassas, VA, USA), MDA-MB-231 cells were cultured in Dulbecco’s Modified Eagle’s Medium (Invitrogen, Carlsbad, CA, USA) supplemented with 10% fetal bovine serum and 1% penicillin/streptomycin, at 37 °C in 5% CO_2_- 95% air. The DU145 tumor cell line of human non-hormone sensitive prostate cancer, was purchased from American Type Culture Collection (ATCC® HTB-81™). DU145 cells were cultured in RPMI medium supplemented with 10% fetal bovine serum and 1% penicillin/streptomycin, at 37 °C in a humidified atmosphere containing 5% CO_2_.

### 3.6. Biological Assay Kit

The cell viability was assessed using standard MTT assay according to the manufacturer’s instructions (MTT cell proliferation assay kit, purchased from TREVIGEN, catalog #4890-025-K, 8405 Helgerman Ct, Gaithersburg, MA, USA).

Analysis of cell proliferation was performed in the presence of different concentration of any synthetized compounds on cell lines seeded in 96-well plates at the density of 1–2 × 10^3^ cells/well in serum-containing media. After 24 h of incubation at 37 °C, cells were treated with increasing concentrations of compounds or vehicle (DMSO; maximum concentration ≤0.5%), following which the cells were cultured in drugs for 48 h, and 10 µL MTT solution (TREVIGEN, Helgerman Ct) was added subsequent to drawing off the medium. The mixture was incubated for an additional 4 h at 37 °C, the absorbance was read at 572 nm in a microplate reader (Biocompare, Italy). Cell viability was expressed as the percentage of proliferated cells compared to cells treated with the different compounds. The same cells were used as control. All experiments were repeated in triplicate [38,39,40,41]. 

### 3.7. Evaluation of the Drug-Membrane Interaction

#### 3.7.1. Liposomes Preparation

Liposomes preparation was carried out following the thin film hydration method [35]. Egg phosphatidylcholine (EPC) was dissolved in chloroform and, then, the obtained solution was evaporated leading to the formation of a lipid film (final lipid concentration 2 mM), which was left under vacuum overnight in order to remove solvent traces. The dried film was hydrated with HEPES buffer (10 mM, I = 0.1 M, pH 7.4) and vortexed at room temperature to get multilamellar vesicles (MLVs). After 30 min at 25 °C, the resultant MLVs suspension was extruded at room temperature 10 times through polycarbonate filters (Nucleopore, Pleasanton, CA, USA, pore size 100 nm) obtaining large unilamellar vesicles (LUVs).

#### 3.7.2. Determination of the Partition Coefficient (Kp) 

The partition coefficient (Kp) of **8** and **4** between liposomes and the aqueous phase was determined by ultrafiltration and using UV-Vis spectrophotometry to quantify drug concentrations in the aqueous phase.

The egg phosphatidylcholine stock suspension was diluted to get a LUVs suspension characterized by an EPC concentration equal to 1000 μM, and contained a constant concentration of the active molecule (40 μM). A reference sample was prepared according to the same experimental protocol but in the absence of liposomes. The obtained samples were incubated at 25 °C for 2 h in dark conditions to reach the partition equilibrium state. Then, the samples were placed in diafiltration tubes (Vivaspin 20 centrifugal concentrator, Sigma-Aldrich, Italy, MWCO 3000 Da) and centrifuged in order to separate the aqueous phase from liposomes. The UV-Vis absorption spectra of the aqueous phases were recorded with a Jasco V-530 UV/Vis spectrometer (Eppendorf, Milan, Italy). The drug concentrations in lipid membrane phase were achieved by subtracting the concentration in the water phase of the tested sample from the concentration in the water phase of the correspondent reference sample prepared in the absence of liposomes. 

Partition coefficients were calculated by the following Equation (1):

(1) $$K_{p}=\frac{n_{m}}{n_{w}}$$

where n_m_ and n_w_ are the number of drug moles distributed on the lipid membrane phase and in the aqueous phase, respectively.

## 4. Synthesis of Ligands

The pro-ligands: 4′-methoxy-phenylfulvene, 6-(3′,4′-dimethoxyphenyl) fulvene were synthesized as reported in the literature and, afterwards, were reduced by a solution of the super-hydride LiBEt**_3_**H.

### 4.1. Synthesis of Group 3 Metal Cyclopentadienyl Complexes

#### 4.1.1. General Procedure

Two separate flasks of the lithium salt of ligands (1.0 mmol) and the appropriate amount of MCl_3_ (1.0 mmol for half-metallocene complexes or 0.5 mmol for metallocene complexes) were weighed in a dry-box. The ligand was dissolved in 8 mL of anhydrous THF and introduced in a dropping funnel, then it was slowly added to a solution of metal halide in 32 mL of THF previously thermostated at −78 °C. The reaction was kept under stirring overnight. The temperature was allowed to rise to room temperature, then the solvent was removed under vacuum and dichloromethane was added the solid. The mixture was filtered on celite, to remove the lithium chloride, and then the solution was dried in vacuum. The products were characterized by NMR analysis. 

#### 4.1.2. Yields and Spectral Data of Scandium Complexes

*[Cp-CH_2_-C_6_H_5_-OCH_3_)]ScCl_2_ [(p-Methoxybenzyl) cyclopentadienyl] scandium dichloride* (**1**) Yield: 95%, ^1^H-NMR (300 MHz, CDCl_3_): 2.80 (dd, 1H, CH_2_), 2.92 (dd, 1H, CH_2_), 3.60 (s, 3H, OCH_3_), 5.98–6.37 (m, 4H, C_5_H_4_), 6.84 (d, 2H, C_6_H_4_), 7.05 (d, 2H, C_6_H_4_). ^13^C-NMR (300 MHz, *d*_6_-DMSO): 42.9 (CH_2_), 55.8 (OCH_3_), 113.3 (C_3′_–C_5′_ of C_6_H_4_), 128.1(C_1′_ of C_6_H_4_), 129.9 (C_2′_–C_6′_ of C_6_H_4_), 131.6 (C_1_ of C_5_H_4_), 132.6–132.9 (C_2_–C_5_ of C_5_H_4_), 134.5–134.8 (C_3_–C_4_ C_5_H_4_), 158.0 (C_4′_ -OCH_3_ of C_6_H_4_). ESI-MS (CH_3_CN, *m*/*z*): 288–290 Dalton [C_13_H_13_OClScNa]^+^. Elemental analysis: found (%): C 51.47; H 4.19. Calc. for C_13_H_13_Cl_2_OSc (%): C 51.86; H 4.35.

*[Cp–CH_2_–C_6_H_4_–(OCH_3_)_2_] ScCl_2_ [(3,4-Dimethoxybenzyl)cyclopentadienyl]scandium dichloride* (**4**). Yield: 33%, ^1^H-NMR (300 MHz, CDCl_3_): 2.77 (dd, 1H, CH_2_), 2.91 (dd, 1H, CH_2_), 3.80 (s, 6H, OCH_3_), 5.87–6.33 (m, 4H, C_5_H_4_), 6.67 (m, 3H, C_6_H_3_). ^13^C-NMR (300 MHz, *d*_6_-DMSO): 33.9 (CH_2_), 54.8 (OCH_3_), 110.6 (C_2′_ of C_6_H_3_), 111.8 (C_5′_ of C_6_H_3_), 112.2 (C_6′_ of C_6_H_3_), 117.9 (C_1′_ of C_6_H_3_), 119.9 (C_1_ of C_5_H_4_), 130.0 (C_2_–C_5_ of C_5_H_4_), 134.9 (C_3_–C_4_ C_5_H_4_), 146.7 (C_4′_–OCH_3_ of C_6_H_3_), 148.3 (C_3′_–OCH_3_ of C_6_H_3_). ESI-MS (CH_3_CN, *m*/*z*): 299.4 Dalton [C_14_H_15_O_2_ScK]^+^. Elemental analysis: found (%): C 50.52; H 4.76. Calc. for C_14_H_15_Cl_2_O_2_Sc (%): C 50.78; H 4.57.

*[Cp-CH_2_-C_6_H_5_-OCH_3_]_2_ScCl bis-[(p-Methoxybenzyl)cyclopentadienyl]scandium chloride* (**7**). Yield: 24%, ^1^H-NMR (300 MHz *d*_6_-DMSO): 2.80 (dd, 2H, CH_2_), 2.92 (dd, 2H, CH_2_), 3.71 (s, 6H, OCH_3_), 5.76–6.38 (m, 8H, C_5_H_4_), 6.83 (d, 4H, C_6_H_4_), 7.10 (d, 4H, C_6_H_4_). ^13^C-NMR (300 MHz, *d*_6_-DMSO): 40.1 (CH_2_), 53.9 (OCH_3_), 112.9 (C_3′_–C_5′_ of C_6_H_4_), 125.7(C_1′_ of C_6_H_4_), 129.3 (C_2′_–C_6′_ of C_6_H_4_), 130.2 (C_1_ of C_5_H_4_), 130.9–131.3 (C_2_–C_5_ of C_5_H_4_), 132.6–133.7 (C_3_–C_4_ of C_5_H_4_), 155.1 (COCH_3_). ESI-MS (CH_3_CN, *m*/*z*): 422.2 Dalton [C_26_H_26_O_2_ScLi]^+^. Elemental analysis: found (%): C 69.79; H 5.12. Calc. for C_26_H_26_ClO_2_Sc (%): C 69.26; H 5.81.

*[Cp-CH_2_-C_6_H_5_-(OCH_3_)_2_]_2_ScCl bis-[(3,4-Dimethoxybenzyl)cyclopentadienyl]scandium chloride* (**10**). Yield: 37%, ^1^H-NMR (400 MHz, CD_2_Cl_2_): 2.93 (dd, 2H, CH_2_), 2.99 (dd, 2H, CH_2_), 3.83 (s, 12H, OCH_3_), 6.17–6.56 (m, 8H, C_6_H_4_), 6.87 (m, 6H, C_6_H_3_). ^13^C-NMR (300 MHz, *d*_6_-DMSO): 36.3 (CH_2_), 55.0 (OCH_3_), 111.9 (C_2′_ of C_6_H_3_), 112.2 (C_5′_ of C_6_H_3_), 114.9 (C_6′_ of C_6_H_3_), 120.4 (C_1′_ of C_6_H_3_), 122.1 (C_1_ of C_5_H_4_), 132.7 (C_2_–C_5_ of C_5_H_4_), 137.2 (C_3_–C_4_ C_5_H_4_), 148.9 (C_4′_–OCH_3_ of C_6_H_3_), 149.2 (C_3′_–OCH_3_ of C_6_H_3_). ESI-MS (CH_3_CN, *m*/*z*): 514.6 Dalton [C_28_H_30_O_4_ScK]^+^. Elemental analysis: found (%): C 65.23; H 5.41. Calc. for C_28_H_30_O_4_ClSc (%): C 65.82; H 5.92. 

#### 4.1.3. Yields and Spectral Data of Yttrium Complexes

*[C_5_H_4_–CH_2_–C_6_H_4_–OCH_3_]YCl_2_ [(p-Methoxybenzyl)cyclopentadienyl]yttrium dichloride* (**2**). Yields: 42%, ^1^H-NMR (300 MHz, *d*_6_-DMSO): 2.80 (dd, 1H, CH_2_), 2.92 (dd, 1H, CH_2_), 3.71 (s, 3H, OCH_3_), 6.22–6.38 (m, 4H, C_5_H_4_), 6.82 (d, 2H, C_6_H_4_), 7.09 (d, 2H, C_6_H_4_). ^13^C-NMR (300 MHz, *d*_6_-DMSO): 42.7 (CH_2_), 54.9 (OCH_3_), 113.7 (C_3′_–C_5′_ of C_6_H_4_), 127.1 (C_1′_ of C_6_H_4_), 129.4 (C_2′_–C_6′_ of C_6_H_4_), 131.4 (C_1_ of C_5_H_4_), 132.5–132.6 (C_2_–C_5_ of C_5_H_4_), 134.0–134.4 (C_3_–C_4_ of C_5_H_4_), 157.5 (COCH_3_). ESI-MS (CH_3_CN, *m*/*z*): 348–350 Dalton [C_13_H_13_ClOYK]^+^. Elemental analysis: found (%): C 45.59; H 3.13. Calc. for C_13_H_13_Cl_2_OY (%): C 45.25; H 3.80.

*[C_5_H_4_–CH_2_–C_6_H_3_(OCH_3_)_2_]YCl_2_ [(3,4-Dimethoxybenzyl)cyclopentadienyl]yttrium dichloride* (**5**). Yield: 46%, ^1^H-NMR (300 MHz, CDCl_3_): 2.82 (dd, 1H, CH_2_), 2.94 (dd, 1H, CH_2_), 3.60 (s, 3H, OCH_3_), 3.71 (s, 3H, OCH_3_), 6.00–6.39 (m, 4H, C_5_H_4_), 6.76 (m, 3H, C_6_H_3_). ^13^C-NMR (300 MHz, *d*_6_-DMSO): 34.7 (CH_2_), 53.5 (OCH_3_), 110.3 (C_2′_ of C_6_H_3_), 111.4 (C_5′_ of C_6_H_3_), 113.3 (C_6′_ of C_6_H_3_), 118.8 (C_1′_ of C_6_H_3_), 120.7 (C_1_ of C_5_H_4_), 130.6 (C_2_–C_5_ of C_5_H_4_), 135.8 (C_3_–C_4_ C_5_H_4_), 147.5 (C_4′_–OCH_3_ of C_6_H_3_), 148.3 (C_3′_–OCH_3_ of C_6_H_3_). ESI-MS (CH_3_CN, *m*/*z*): 238.2 Dalton [C_14_H_15_O_2_YNa]^+^. Elemental analysis: found (%): C 44.21; H 4.54. Calc. for C_14_H_15_Cl_2_O_2_Y (%): C 44.83; H 4.03.

*[Cp-CH_2_-C_6_H_5_-OCH_3_]_2_YCl bis-[(p-Methoxybenzyl)cyclopentadienyl]yttrium chloride* (**8**). Yield: 21%, ^1^H-NMR (300 MHz, *d*_6_-DMSO): 2.80 (dd, 2H, CH_2_), 2.92 (dd, 2H, CH_2_), 3.70 (s, 6H, OCH_3_), 5.99–6.38 (m, 8H, C_5_H_4_), 6.84 (d, 4H, C_6_H_4_), 7.10 (d, 4H, C_6_H_4_). ^13^C-NMR (300 MHz, *d*_6_-DMSO): 40.8 (CH_2_), 54.3 (OCH_3_), 113.1 (C_3′_–C_5′_ of C_6_H_4_), 125.9(C_1′_ of C_6_H_4_), 129.3 (C_2′_–C_6′_ of C_6_H_4_), 130.4 (C_1_ of C_5_H_4_), 131.1–131.6 (C_2_–C_5_ of C_5_H_4_), 133.6–134.0 (C_3_–C_4_ of C_5_H_4_), 156.1 (COCH_3_). ESI-MS (CH_3_CN, *m*/*z*): 482.9 Dalton [C_26_H_26_O_2_YNa]^+^. Elemental analysis: found (%): C 62.89; H 5.34. Calc. for C_26_H_26_ClO_2_Y (%): C 63.11; H 5.30.

*[Cp-CH_2_-C_6_H_5_-(OCH_3_)_2_]_2_YCl bis-[(3,4-Dimethoxybenzyl)cyclopentadienyl]yttrium chloride* (**11**). Yield: 36%, ^1^H-NMR (400 MHz, *d*_6_-DMSO): 2.88 (dd, 2H, CH_2_), 3.02 (dd, 2H, CH_2_), 3.79 (s, 12H, OCH_3_), 6.08–6.48 (m, 8H, C_6_H_4_), 6.93 (d, 4H, C_6_H_3_), 7.19 (d, 2H, C_6_H_3_). ^13^C-NMR (300 MHz, *d*_6_-DMSO): 35.7 (CH_2_), 54.8 (OCH_3_), 111.3 (C_2′_ of C_6_H_3_), 111.8 (C_5′_ of C_6_H_3_), 114.2 (C_6′_ of C_6_H_3_), 119.0 (C_1′_ of C_6_H_3_), 121.9 (C_1_ of C_5_H_4_) , 131.6 (C_2_–C_5_ of C_5_H_4_), 136.8 (C_3_–C_4_ C_5_H_4_), 148.5 (C_4′_–OCH_3_ of C_6_H_3_), 148.8 (C_3′_–OCH_3_ of C_6_H_3_). ESI-MS (CH_3_CN, *m*/*z*): 526.1 Dalton [C_28_H_30_O_4_YLi]^+^. Elemental analysis: found (%): C 60.11; H 5.92. Calc. for C_28_H_30_O_4_ClY (%): C 60.61; H 5.45.

#### 4.1.4. Yields and Spectral Data of Neodymium Complexes

*[C_5_H_4_–CH_2_–C_6_H_4_–OCH_3_]NdCl_2_ [(p-Methoxybenzyl)cyclopentadienyl]neodymium dichloride* (**3**). Yield: 49%, ^1^H-NMR (250 MHz. *d*_6_-DMSO): 2.80 (dd, 1H, CH_2_), 2.93 (dd, 1H, CH_2_), 3.71 (s, 3H, OCH_3_), 5.99–6.38 (m, 4H, C_5_H_4_), 6.85 (d, 2H, C_6_H_4_), 7.11 (d, 2H, C_6_H_4_). ^13^C-NMR (300 MHz, *d*_6_-DMSO): 40.6 (CH_2_), 53.8 (OCH_3_), 112.7 (C_3′_–C_5′_ of C_6_H_4_)), 126.1(C_1′_ of C_6_H_4_), 127.3 (C_2′_–C_6′_ of C_6_H_4_), 130.2 (C_1_ of C_5_H_4_), 131.2–131.9 (C_2_–C_5_ of C_5_H_4_), 133.9–134.0 (C_3_–C_4_ of C_5_H_4_), 156.8 (COCH_3_). ESI-MS (CH_3_CN, *m*/*z*): 368.2 Dalton [C_13_H_13_ONdK]^+^. Elemental analysis: found (%): C 38.68; H 3.41. Calc. for C_13_H_13_Cl_2_ONd (%): C 39.00; H 3.27.

*[C_5_H_4_–CH_2_–C_6_H_3_ (OCH_3_)_2_]NdCl_2_ [(3,4-Dimethoxybenzyl)cyclopentadienyl]neodymium dichloride* (**6**). Yield: 30%, ^1^H-NMR (250 MHz, CDCl_3_): 2.79 (dd, 1H, CH_2_), 2.92 (dd, 1H, CH_2_), 3.79 (s, 6H, OCH_3_), 5.93–6.35 (m, 4H, C_5_H_4_), 6.68 (m, 3H, C_6_H_3_). ^13^C-NMR (300 MHz, *d*_6_-DMSO): 35.7 (CH_2_), 54.5 (OCH_3_), 110.1 (C_2′_ of C_6_H_3_), 111.3 (C_5′_ of C_6_H_3_), 113.2 (C_6′_ of C_6_H_3_), 117.9 (C_1′_ of C_6_H_3_), 121.6 (C_1_ of C_5_H_4_), 130.5 (C_2_–C_5_ of C_5_H_4_), 134.6 (C_3_–C_4_ C_5_H_4_), 146.2 (C_4′_–OCH_3_ of C_6_H_3_), 147.3 (C_3′_–OCH_3_ of C_6_H_3_). ESI-MS (CH_3_CN, *m*/*z*): 418–420 Dalton [C_14_H_15_O_2_ClNdNa]^+^. Elemental analysis: found (%): C 39.61; H 3.92. Calc. for C_14_H_15_Cl_2_O_2_Nd (%): C 39.07; H 3.51.

*[Cp-CH_2_-C_6_H_5_-OCH_3_]_2_NdCl bis-[(p-Methoxybenzyl) cyclopentadienyl] neodymium chloride* (**9**). Yield: 23%, ^1^H-NMR (300 MHz, *d*_6_-DMSO): 2.86 (dd, 2H, CH_2_), 2.97 (dd, 2H, CH_2_), 3.79 (s, 6H, OCH_3_), 6.14–6.41 (m, 4H, C_5_H_4_), 6.85 (d, 4H, C_5_H_4_), 7.10 (d, 4H, C_5_H_4_). ^13^C-NMR (300 MHz, *d*_6_-DMSO): 41.8 (CH_2_), 53.3 (OCH_3_), 112.1 (C_3′_–C_5′_ of C_6_H_4_), 124.9(C_1′_ of C_6_H_4_), 128.3 (C_2′_–C_6′_ of C_6_H_4_), 129.4 (C_1_ of C_5_H_4_), 130.1–130.6 (C_2_–C_5_ of C_5_H_4_), 132.6–133.0 (C_3_–C_4_ of C_5_H_4_), 155.1 (COCH_3_). ESI-MS (CH_3_CN, *m*/*z*): 521.3 Dalton [C_26_H_26_O_2_NdLi]^+^. Elemental analysis: found (%): C 56.11; H 4.52. Calc. for C_26_H_26_ClO_2_Nd (%): C 56.76; H 4.76.

*[Cp-CH_2_-C_6_H_5_-(OCH_3_)_2_]_2_NdCl bis-[(3,4-Dimethoxybenzyl)cyclopentadienyl]neodymium chloride* (**12**). Yield: 55%, ^1^H-NMR (400 MHz, *d*_6_-DMSO): 2.79 (dd, 2H, CH_2_), 2.92 (dd, 2H, CH_2_), 3.80 (s, 12H, OCH_3_), 5.94–6.346 (m, 8H, C_6_H_4_), 6.69 (m, 6H, C_6_H_3_). ^13^C-NMR (300 MHz, *d*_6_-DMSO): 36.7 (CH_2_), 55.8 (OCH_3_), 112.3 (C_2′_ of C_6_H_3_), 112.8 (C_5′_ of C_6_H_3_), 113.2 (C_6′_ of C_6_H_3_), 120.0 (C_1′_ of C_6_H_3_), 122.9 (C_1_ of C_5_H_4_), 132.6 (C_2_–C_5_ of C_5_H_4_), 137.8 (C_3_–C_4_ C_5_H_4_), 149.5 (C_4′_–OCH_3_ of C_6_H_3_), 150.0 (C_3′_–OCH_3_ of C_6_H_3_). ESI-MS (CH_3_CN, *m*/*z*): 613.2 Dalton [C_28_H_30_O_4_NdK]^+^. Elemental analysis: found (%): C 55.19; H 4.42. Calc. for C_28_H_30_O_4_ClNd (%): C 55.11; H 4.96.

## 5. Statistical Analysis

The MTT assay data were statistically analyzed with descriptive statistics software (Graphpad®) to assess the means and standard deviation (the values are reported in figures as percentage of cell growth). The IC_50_ has been evaluated adopting a statistical nonlinear regression model, applying a polynomial fitting curve on the base of our empiric data. The goodness of fit has been evaluated through the definition of the coefficient of determination (R^2^) that results in the range of 0.789 < R^2^ < 0.987, considering all of our models.

## 6. Conclusions

We reported the synthesis process and characterization of new scandium (III), yttrium (III), and neodymium (III) chloride complexes with substituted cyclopentadienyl ancillary ligands, highlighting their activity for the treatment of solid tumors, such as triple negative breast cancer (TN) and prostate cancer. The ligands were chosen among those that showed ability to stabilize analogous titanium complexes with significant antitumor activity. 

The anti-proliferative effect of novel synthesized compounds were evaluated on MDA.MB231, human triple negative breast cancer cells, and DU145, human non-hormone sensitive prostate cancer cells. Our results highlight three compounds with the best inhibitory activity on both cell lines:

(**1**) *[(p-methoxybenzyl)cyclopentadienyl]scandium dichloride;*

(**5**) *[(3,4-dimethoxybenzyl)cyclopentadienyl]yttrium dichloride;*

(**8**) *bis-[(p-methoxybenzyl)cyclopentadienyl]yttrium chloride;*

Instead there is only one compound that imparts a strong effect only on the DU145 cell line:

(**3**) *[(p-methoxybenzyl)cyclopentadienyl]neodymium dichloride.*

The compounds based on yttrium (**5**) and (**8**) show very interesting results, being active at very low concentration (IC_50_ < 5 µM), probably due to their ability to induce apoptosis. The scandium complex (**1**) is the only truly interesting one among those based on this metal, whereas those based on neodymium do not show remarkable activity.

According to our data on the inhibitory activity of cisplatin on DU145 cell lines (IC_50_ ≈ 2 μM) and MDAMB231 (IC_50_ ≈ 5 μM) we can affirm that our best compounds **1**, **8**, **3** have a similar inhibitory concentration 50 (IC_50_).

The interaction of the synthesized complexes **8** and **4** with biological membranes was also investigated and our results highlighted a significant membrane permeability, this feature could allow the adoption of these molecules in future studies.

The results have been tentatively rationalized on the basis of electronic and steric considerations. 

## Figures and Tables

**Table 1:** Synthesized compounds **1**–**12**.

Compound	R	M	Name
**1**	H	Sc	*[(p-methoxybenzyl)cyclopentadienyl]scandium dichloride*
**2**	H	Y	*[(p-methoxybenzyl)cyclopentadienyl]yttrium dichloride*
**3**	H	Nd	*[(p-methoxybenzyl)cyclopentadienyl]neodymium dichloride*
**4**	-OCH_3_	Sc	*[(3,4-dimethoxybenzyl)cyclopentadienyl]scandium dichloride*
**5**	-OCH_3_	Y	*[(3,4-dimethoxybenzyl)cyclopentadienyl]yttrium dichloride*
**6**	-OCH_3_	Nd	*[(3,4-dimethoxybenzyl)cyclopentadienyl]neodymium dichloride*
**7**	H	Sc	*bis-[(p-methoxybenzyl)cyclopentadienyl]scandium chloride*
**8**	H	Y	*bis-[(p-methoxybenzyl)cyclopentadienyl]yttrium chloride*
**9**	H	Nd	*bis-[(p-methoxybenzyl)cyclopentadienyl]neodymium chloride*
**10**	-OCH_3_	Sc	*bis-[(3,4-dimethoxybenzyl)cyclopentadienyl]scandium chloride*
**11**	-OCH_3_	Y	*bis-[(3,4-dimethoxybenzyl)cyclopentadienyl]yttrium chloride*
**12**	-OCH_3_	Nd	*bis-[(3,4-dimethoxybenzyl)cyclopentadienyl]neodymium chloride*

**Table 2:** Effect of complexes **1**–**12** on MDA.MB231, human triple negative breast cancer cells, and DU145, prostate cancer cell line.

MDA.MB231			DU145		
Scandium	Yttrium	Neodymium	Scandium	Yttrium	Neodymium
Compounds IC_50_ (µM)	Compounds IC_50_ (µM)	Compounds IC_50_ (µM)	Compounds IC_50_ (µM)	Compounds IC_50_ (µM)	Compounds IC_50_ (µM)
**1** <10	**5** <5	**3** 12	**1** <10	**5** <5	**3** 12
**4** <20	**8** <5	**6** 50	**4** <20	**8** <5	**6** 50
**7** 20	**11** <50		**7** <20	**11** <50	

## Supplementary Materials

Supplementary materials are available online.
