# Supplementary material for: Synthesis and Antitumor Activity of New Group 3 Metallocene Complexes
Source: Molecules. 2017 Mar 28;22(4):526. doi: 10.3390/molecules22040526 (PMC6154682; doi:10.3390/molecules22040526)
Supplement: Supplementary file 1 [file molecules-22-00526-s001.pdf]

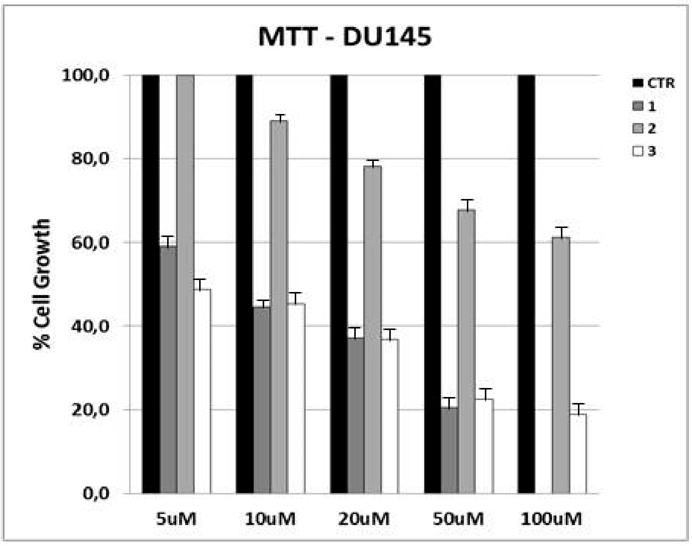
MTT - DU145
% Cell Growth
CTR
1
2
3
5uM
10uM
20uM
50uM
100uM

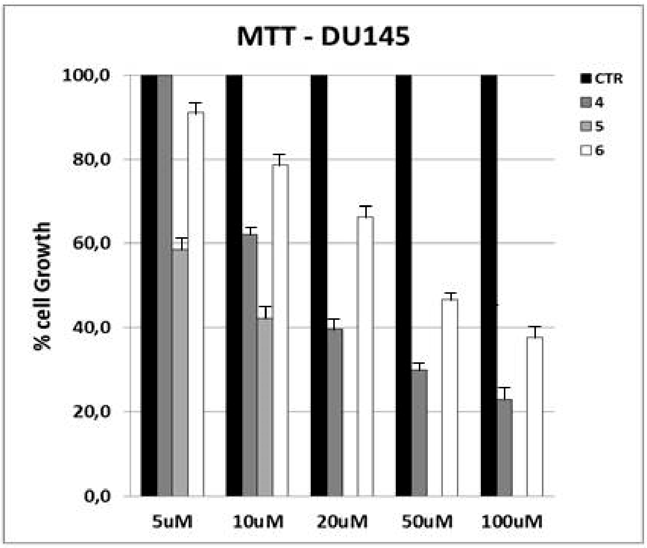
MTT - DU145
% cell Growth
CTR
4
5
6
5uM
10uM
20uM
50uM
100uM

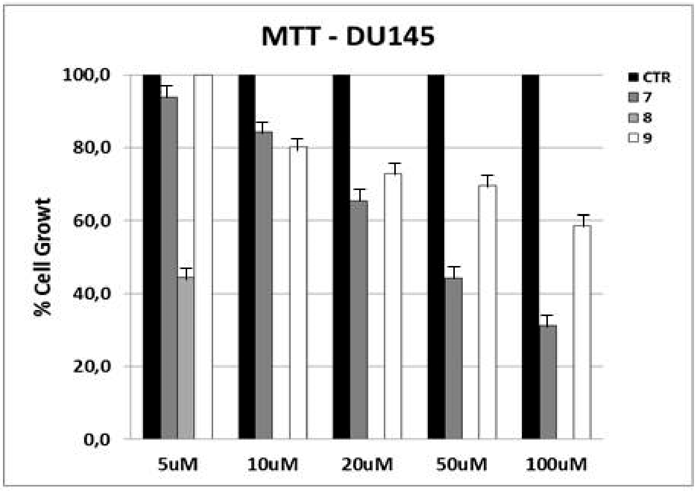
MTT - DU145
% Cell Growt
CTR
7
8
9
5uM
10uM
20uM
50uM
100uM

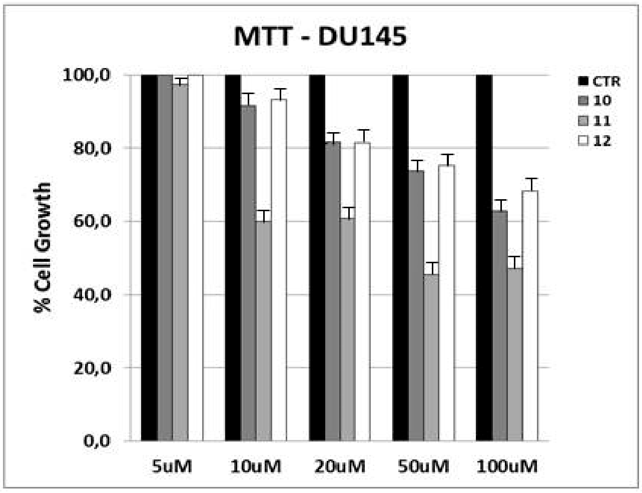
MTT - DU145
% Cell Growth
CTR
10
11
12
5uM
10uM
20uM
50uM
100uM

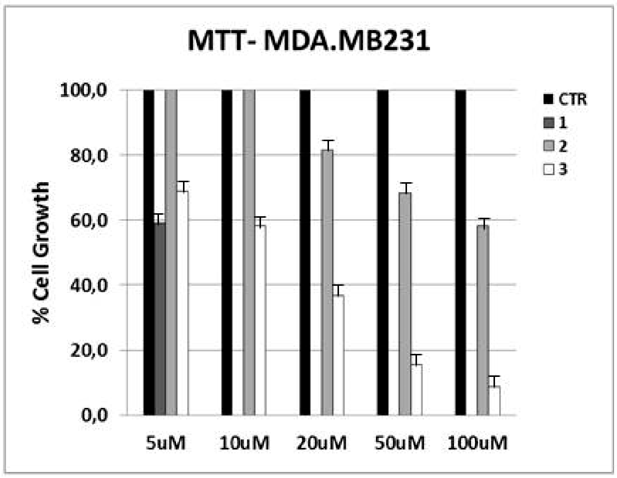
MTT- MDA.MB231
% Cell Growth
100,0
80,0
60,0
40,0
20,0
0,0
5uM
10uM
20uM
50uM
100uM
CTR
1
2
3

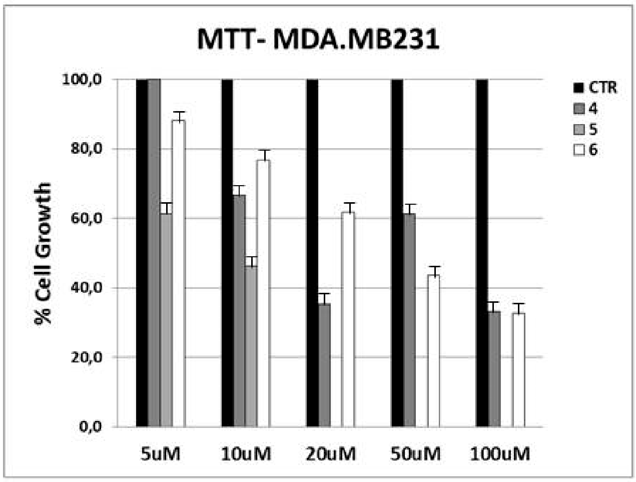
MTT- MDA.MB231
% Cell Growth
100,0
80,0
60,0
40,0
20,0
0,0
5uM
10uM
20uM
50uM
100uM
CTR
4
5
6

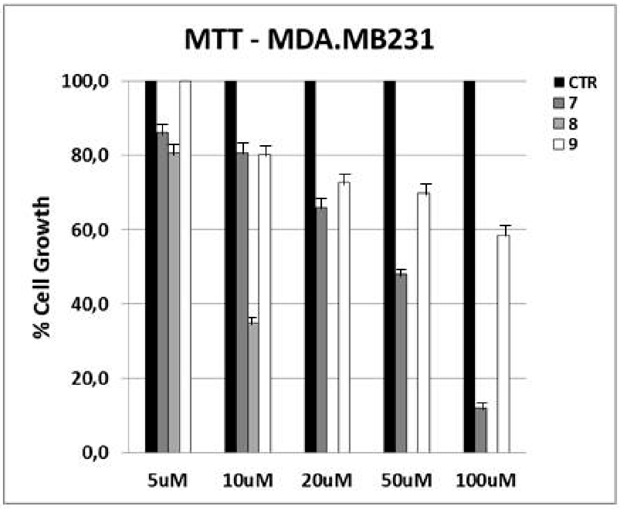
MTT - MDA.MB231
% Cell Growth
100,0
80,0
60,0
40,0
20,0
0,0
5uM
10uM
20uM
50uM
100uM
CTR
7
8
9

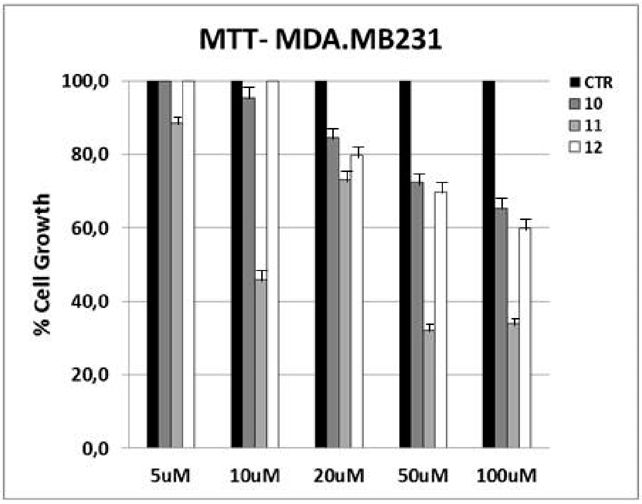
MTT- MDA.MB231
% Cell Growth
100,0
80,0
60,0
40,0
20,0
0,0
5uM
10uM
20uM
50uM
100uM
CTR
10
11
12

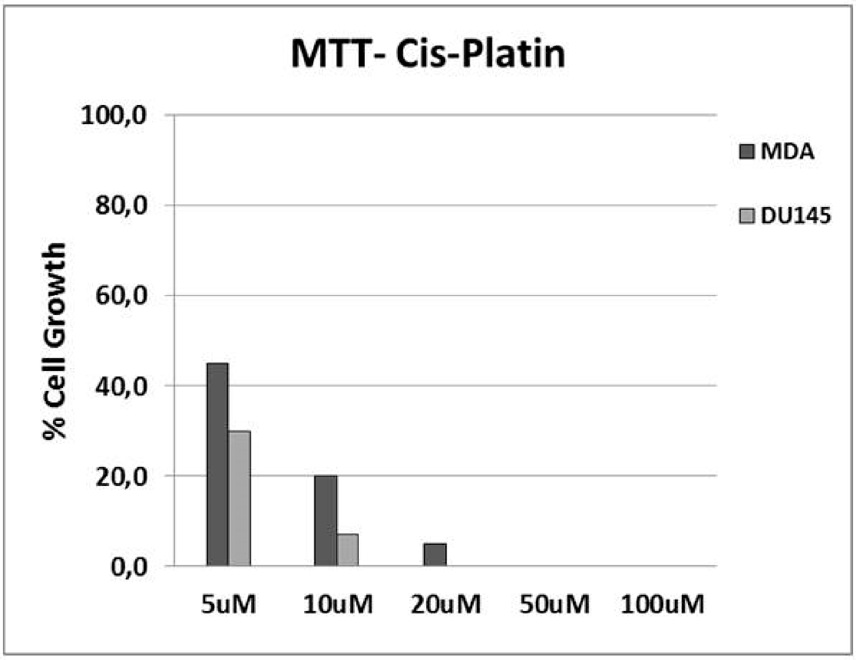

Supplementary Information: *Evaluation of the cytotoxic activity of compounds was evaluated as IC50 (µM) responses from 5µM to 100µM of compounds 1÷12 in MDA.MB231, human Triple Negative Breast cancer (a), and DU145, human Prostate cancer not hormone sensitive (b), Cisplatin IC50 on our cancer cell lines (c), defined by the MTT assay. Cell growth was expressed as percentage of cells treated with different concentrations, distinct compounds respect to cells treated for 48 hours.*
